# Correlation between posterior teeth loss and temporomandibular joint disorder symptoms in adult patients

**DOI:** 10.12688/f1000research.161006.1

**Published:** 2025-04-11

**Authors:** Batool Hussein Ayaz, May Wathiq Al-Khudhairy

**Affiliations:** 1 College of Dentistry, Riyadh Elm University, Riyadh, Saudi Arabia

**Keywords:** temporomandibular joint disorder; tooth loss; posterior teeth

## Abstract

**Background/Objectives:**

The relation between TMD and posterior tooth loss is still up for debate and is a topic of constant discussion. The present study aimed to find a correlation between posterior teeth loss and TMJ disorder symptoms in adult patients.

**Methods:**

A total of fifty patients were selected for the study. First, intra-oral dental examination recorded all missing posterior teeth, the teeth numbers, type of missing teeth (except third molars). All existing teeth are examined during the clinical examination to identify any odontogenic causes for pain if present. Second, patients were asked to fill in the Arabic-translated form of the DC/TMD Axis I symptom questionnaire. Third, the DC/TMD Axis II protocol was applied to each patient and the examination form was completed by the examiner during the clinical examination.

**Results:**

Disc displacement with reduction is more likely to cause headache, pain, and clicking. A statistically significant positive correlation was found between age and the number of missing teeth. Furthermore, a statistically significant negative correlation was found between age and maximum unassisted mouth opening, and the number of missing teeth and maximum unassisted mouth opening. Logistics regression analysis showed clicking was significantly associated and 13.8 (OR) times more likely to have TMD.

**Conclusions:**

The current study reported that patients with TMD are more likely to have pain, headache, clicking, and a decrease in maximum mouth opening. There was a correlation between clicking and TMJ disorder, and the number of tooth loss and TMJ disorder.

## Introduction

A variety of clinical symptoms affecting the temporomandibular joint (TMJ), the muscles of mastication, or the related orofacial tissues are grouped under the name "temporomandibular disorders" (TMD). Specific symptoms, including headaches, neck pain, abnormal noises during jaw movements, pain or sensitivity in the chewing muscles or TMJ region, limited or incoherent movements, and an inappropriate relationship between jaw positions characterize it. ^1^ It is currently unclear what causes TMD. ^2^ TMD has been linked to several conditions, including bruxism, tooth grinding or clenching, ^3^ osteoarthrosis, abnormal occlusion, ^4^ tooth wear, ^5^ non-working-side occlusal interferences, ^6^ limited mandibular movements, ^7^ partial loss of teeth, ^8^ masseter muscle activity, ^9^ osteoarthritis, ^10^ and reduced maximum bite force. ^11^ The connection between TMD and posterior tooth loss is still up for debate and is a topic of constant discussion.

TMD is more common in people who lose their posterior teeth, particularly young women, with less missing posterior teeth in more quadrants. ^12^ However, TMD is not associated with malocclusion or the loss of five or more posterior teeth, ^13^ and there is no relation between the number of lost teeth and TMD. ^14^ There is inconsistency in the literature because some research found no relation between TMD and the number of absent posterior teeth. ^15– 17^ Other authors, however, have claimed that losing molar support was associated with the presence and severity of osteoarthrosis ^18– 20^ or with TMD. ^21– 24^ The current study aimed to find a correlation between posterior teeth loss and symptoms of TMJ disorder in adult patients.

## Methods

The present cross-sectional analytical study was carried out at Riyadh Elm University's prosthodontics department in Riyadh, Kingdom of Saudi Arabia. The Institutional Review Board of Riyadh Elm University in Riyadh, Kingdom of Saudi Arabia, gave its approval to the study (FPGRP/2021/574/527) in December 2021. The study was carried out between January and April of 2022. Participants had to be older than eighteen, have maxillary and mandibular front teeth, and have two or more posterior teeth (molars and premolars) missing for longer than six months, with the exception of the third molars. Subjects wearing removable partial dentures who had experienced traumatic tooth loss (e.g., a car accident, gunshot, maxillofacial surgery, etc.), those with a history of diagnosed and treated symptomatic TMD, and those currently diagnosed with fibromyalgia, trigeminal neuralgia, or on medication were excluded. Samples were chosen using convenience sampling. For the investigation, 50 samples in total that satisfied the inclusion requirements were chosen. Before the trial started, each participant gave their informed consent.

Since DC/TMD Axis-I and Axis-II protocols have a high dependability index value and are regarded as the gold standard, all relevant data pertaining to TMD was collected using them. ^25^ An international version of the clinical examination and a symptom questionnaire are part of the Axis-I protocol. Psychosocial state and pain-related impairment are assessed as part of the Axis II methodology. The decision tree and diagnostic criteria were used to make the diagnosis. ^26^

### Clinical examination

An intraoral dental examination was conducted first, and all missing posterior teeth were noted along with their numbers and kind (molar, premolar, except third molars). During the clinical examination, every tooth that was in place was inspected to determine whether there were any odontogenic reasons for pain. Second, the Arabic-translated version of the DC/TMD Axis I symptom questionnaire ( www.rdc-tmdinternational.org) was given to the patients. This questionnaire covered symptoms such as headache, pain, jaw joint sounds, closed locking of the jaw, and open locking of the jaw. Third, during the clinical examination, the examiner filled out the examination forms and administered the DC/TMD Axis II procedure to each patient.

The examiner examined both TMJ simultaneously on both sides of the face using fingertips, by touching the joints (lateral pole) and adjacent area at 5 to 6 points spaced approximately .5 cm apart (around lateral pole). 0.5 kg pressure was applied on the lateral pole, and 1 kg pressure was applied around the lateral pole. Since too much pressure applied during the examination leads to unreliable results, the force applied by the examiner during palpation is measured using a device called a pressure Algometer.

All the clinical examinations were done by only one examiner who is trained in DC/TMD clinical examination by an orofacial pain specialist to avoid inter-examiner variation. Intra- examiner variability was done for every 5 cases and one case was repeated by the examiner to see if there is any variation. To avoid the improper diagnosis all the diagnosis was done by an orofacial pain specialist. While assessing the pain of muscles and joints. DC/TMD protocol was used to assess TMD symptoms following the Diagnostic Criteria for Temporomandibular Disorders (DC/TMD), according to guidelines of the International Network for Orofacial Pain and Related Disorders ( https://ubwp.buffalo.edu/rdc-tmdinternational/tmd-assessment diagnosis/dc-tmd/).

### Statistical analysis

The Statistical Package for Social Science (SPSS), version 20 (IBM SPSS Statistics for Windows, IBM Corp., Armonk, NY, USA), was used for all statistical analyses. The mean, standard deviation, frequency, and percentage were among the descriptive statistics that were computed. The Mann-Whitney U test was used for comparison, while the Pearson Chi-Square and Fisher's Exact tests were used for association. We used Spearman's rho to perform correlations. Logistics regression analysis was used for multivariate analysis. Statistical significance was set as p-value of less than 0.05.

## Results

The mean age of the subject was 41.5 *±*6.3 years. The mean number of missing teeth, duration of missing teeth, and maximum unassisted opening of mouth were 3.4 *±*1.3, 5 *±*2.9, and 49.2 *±*5.0, respectively.

The mean maximum mouth opening was higher with no headache ( *50.8±4.2*) (p<0.05). On the other hand, the mean age ( *42.8±7.5*) (p>0.05), number of missing teeth ( *3.8±1.4*)(p>0.05), and duration of tooth loss ( *6.6±3.5*)(p<0.05) were higher with the headache ( Table 1). Disc displacement with reduction is more likely to cause a headache (p<0.05) ( Table 2). The maximum mouth opening was higher with no pain ( *50.0±4.5*) (p<0.05). On the other hand, the mean age ( *42.8±5.9*) (p>0.05), number of missing teeth ( *4.4±1.5*) (p>0.05), and duration of tooth loss ( *9.2±3.7*) (p<0.05) were higher with pain ( Table 3). Displacement with reduction is more likely to cause pain (p<0.05) ( Table 4). The maximum mouth opening was higher with no clicking ( *51.8±2.4*) (p<0.05). On the other hand, the mean age clicking ( *46.2±5.6*) (p<0.05), number of missing teeth clicking ( *4.8±1.0*) (p<0.05), and duration of tooth loss ( *8.4±2.5*) were higher with clicking (p<0.05) ( Table 5). Disc displacement with reduction and reported TMJ click (p<0.05) is more likely to have clicking (p<0.05) ( Table 6).

**Table 1.** Comparison of maximum mouth opening, age, number of missing teeth, and duration of tooth loss with headache.

	Headache Mean±SD		p-value
	Yes	No	
**Maximum mouth opening**	45.8±5.1	50.8±4.2	0.001 *
**Age**	42.8±7.5	40.8±5.7	0.306
**Number of missing teeth**	3.8±1.4	3.2±1.2	0.192
**Duration of tooth loss**	6.6±3.5	4.3±2.3	0.029 *

* Statistical significance at p<0.05.

**Table 2.** Association between TMJ disorders and headache.

		Headache frequency (Percent)		p-value
		Yes	No	
**TMJ disorder**	*None*	8 (22.9)	27 (77.1)	0.049 *
	*Disc displacement with reduction*	8 (53.3)	7 (46.7)	
**Reported TMJ click**	*Yes*	2 (33.3)	4 (66.7)	1.000
	*No*	14 (31.8)	30 (68.2)	

* Statistical significance at p<0.05.

**Table 3.** Comparison of maximum mouth opening, age, number of missing teeth, and duration of tooth loss with pain.

	Pain Mean±SD		p-value
	Yes	No	
**Maximum mouth opening**	41.4±1.9	50.0±4.5	0.000 *
**Age**	42.8±5.9	41.3±6.4	0.624
**Number of missing teeth**	4.4±1.5	3.3±1.2	0.068
**Duration of tooth loss**	9.2±3.7	4.6±2.5	0.000 *

* Statistical significance at p<0.05.

**Table 4.** Association between TMJ disorders and pain.

		Pain Frequency (Percent)		p-value
		Yes	No	
**TMJ disorder**	*None*	1 (2.9)	34 (97.1)	0.024 *
	*Disc displacement with reduction*	4 (26.7)	11 (73.3)	
**Reported TMJ click**	*Yes*	1 (16.7)	5 (83.3)	1.000
	*No*	7 (15.9)	37 (84.1)	

* Statistical significance at p<0.05.

**Table 5.** Comparison of maximum mouth opening, age, number of missing teeth, and duration of tooth loss with clicking.

	Clicking Mean±SD		p-value
	Yes	No	
**Maximum mouth opening**	43.9±4.8	51.8±2.4	0.000 *
**Age**	46.2±5.6	39.0±5.3	0.000 *
**Number of missing teeth**	4.8±1.0	2.7±0..7	0.000 *
**Duration of tooth loss**	8.4±2.5	3.3±1.0	0.000 *

* Statistical significance at p<0.05.

**Table 6.** Association between TMJ disorders and clicking.

		Clicking Frequency (Percent)		p-value
		Yes	No	
**TMJ disorder**	*None*	3 (8.6)	32 (91.4)	0.000 *
	*Disc displacement with reduction*	14 (93.3)	1 (6.7)	
**Reported TMJ click**	*Yes*	6 (54.5)	5 (45.5)	0.000 *
	*No*	0 (0.0)	39 (100.0)	

* Statistical significance at p<0.05.

A statistically significant positive correlation was found between age and the number of missing teeth (r=0.607) (p<0.05). Furthermore, a statistically significant negative correlation was found between age and maximum unassisted mouth opening (r=-0.402) (p<0.05); and the number of missing teeth and maximum unassisted mouth opening (r=-0.502) (p<0.05) ( Table 7). The mean number of missing teeth was statistically significantly higher in TMJ disorder (Disc displacement with reduction) (4.7 **±** 1.0) (p<0.05) ( Table 8).

**Table 7.** Correlation between various variables.

Variables	Correlation coefficient	p-value
Age-Number of missing teeth	0.607	0.000 *
Age-Maximum unassisted mouth opening	-0.402	0.004 *
Number of missing teeth-Maximum unassisted mouth opening	-0.502	0.000 *

* Statistical significance at p<0.05.

**Table 8.** Comparison of mean (sd) number of missing teeth with TMJ disorder.

TMJ Disorder	Mean±SD number of missing teeth	p-value
None	2.8 (0.9)	0.000 *
Disc displacement with reduction	4.7 (1.0)	

* Statistical significance at p<0.05.

The number of missing teeth showed OR=2.620 times higher risk of TMD (variable) as well as pain showed OR=6.089 times higher (p>0.05). Whereas, clicking was significantly associated and OR=13.8 times more likely to have TMD (variable) (p<0.05) ( Table 9). The pain was OR=6.021 times more likely associated with TMD (p>0.05). Whereas, clicking showed a significant association with the TMD (variable) (p<0.05) ( Table 10).

**Table 9.** Logistics regression analysis of the number of missing teeth with the TMD.

Model variables	Adj. OR	95% C.I.		p-value
		Lower bound	Upper bound	
**Number of missing teeth**	2.62	0.699	9.812	0.153
**Pain**				
Yes	6.089	0.071	525.156	0.427
No	1			
**Headache**				
Yes	0.97	0.097	9.73	0.979
No	1			
**Clicking sound**				
Yes	13.86	1.051	182.857	0.046 *
No	1			

* Statistical significance at p<0.05.

**Table 10.** Logistics regression analysis of symptoms together with the TMD.

Model variables	Adj. OR	95% C.I.		p-value
		Lower bound	Upper bound	
**Pain**				
Yes	6.021	0.143	253.296	0.347
No	1			
**Headache**				
Yes	0.708	0.074	6.803	0.765
No	1			
**Clicking**				
Yes	67.22	9.014	501.267	0.001 *
No	1			

* Statistical significance at p<0.05.

## Discussion

The relationship between multiple posterior teeth missing and TMJ disorders always remains controversial. In addition, signs and symptoms like headache and muscular pain, TMJ sound, and restricted mouth opening may be some of the signs or symptoms associated with TMD disorders. Thus, the present study was planned to assess the correlation between posterior teeth loss and TMJ disorder symptoms in adult patients. The current study showed that patients with TMD (TMJ disc displacement with reduction) are more likely to have pain, headache, and TMJ clicking with statistically significant differences. A study by Aggarwal et al. is in agreement with the present study findings which showed that headaches occur more frequently in patients with TMD symptoms (27.4% vs 15.2%). ^27^ In line with the current investigation, another study by Derwich et al. found that TMJ reciprocal clicking is a common clinical sign of disc displacement with reduction and one of the most prevalent forms of TMJ internal derangements. ^28^

Furthermore, the present study showed that maximum mouth opening was greater with (no pain, no headache, and no TMJ clicking) with statistically significant differences. There are many previous reports relating to mouth opening and headaches. Schokker et al. found a close relationship between recurrent headaches and craniomandibular disorders, including the opening of the mouth. ^29^ Calixte et al. also reported less headaches with improved mouth-opening. ^30^ The degree of mouth opening and the quantity of auditory symptoms are associated with the severity of TMD, according to yet another study report by Kitsoulis et al. ^31^ The present study showed that the number of missing teeth and the age increased as the maximum unassisted mouth opening decreased. This result is consistent with a study by Sawair et al. who showed that wide unassisted maximum mouth opening was associated with less risk of tooth loss and preservation of the third molar. ^32^

The present study found that the number of missing teeth was higher with disc displacement with a reduction with a statistically significant difference. According to a study by Tallents et al., the TMJ components become overloaded when posterior teeth are absent. The lack of posterior teeth has been hypothesized and experimentally demonstrated to cause mandibular overclosure, which would cause the condyles to shift from their typical centric position in the TMJ, resulting in joint dislocation. ^24^ Another study found that articular eminence in younger people and the onset of lesions on the load-bearing articular surfaces of the condyle were both influenced by loss of molar support. ^33^ Thus, it appears that most of the study reports are in accordance with the present study. The loss of posterior teeth is expected to bring similar changes in the TMJ joints. Missing posterior teeth appears to be responsible for the changes seen in the TMJ. ^34^

The evaluation in this study was conducted using the DC/TMD axis I and II, which is very pain-oriented, and (dis)function is not given much attention. Furthermore, the classification process, which is more subjective in nature, still heavily relies on palpation and operationalized pressure on the tissues of the masticatory muscles and the TMJs. Overdiagnosis (myofascial pain) and overtreatment while adhering to this classification are the outcomes. However, there was also evidence of the potential for underdiagnosis when using the RDC/TMD criteria. Therefore, it is necessary to implement a balance between these two. In the field of dentistry, the link between tooth loss and temporomandibular disorders is contentious because there may be more than one cause for these issues. Future research will need to evaluate factors like age, gender, and estrogen level. Additionally, evaluating research on secondary occlusion contact feature changes after posterior tooth loss, the effects of these secondary occlusion modifications, and the possible advantages of rectifying such occlusal abnormalities would be interesting.

## Conclusions

Pain, headaches, clicking, and a reduction in maximum mouth opening are more common in TMD patients. There is a relation between TMJ disorders and clicking; and TMJ disorders and the number of tooth loss.

## Author contributions

Conceptualization, B.H.A. and M.W.A.; methodology, B.H.A. and M.W.A; software, B.H.A. and M.W.A; validation, B.H.A. and M.W.A; formal analysis, B.H.A. and M.W.A; investigation, B.H.A. and M.W.A; resources, B.H.A. and M.W.A; data curation, B.H.A. and M.W.A; writing—original draft preparation, B.H.A. and M.W.A; writing—review and editing, B.H.A. and M.W.A; visualization, B.H.A. and M.W.A; supervision, B.H.A. and M.W.A; project administration, B.H.A. and M.W.A; funding acquisition, B.H.A. and M.W.A. All authors have read and agreed to the published version of the manuscript.

## Ethics and consent statement

The study was approved by the Institutional Review Board of Riyadh Elm University, Riyadh, Kingdom of Saudi Arabia (FPGRP/2021/574/527) on 25 December 2021. Written and informed consent was obtained from all subjects involved in the study.

## Data Availability

Figshare.- Correlation between Posterior Teeth Loss and Temporomandibular Joint Disorder Symptoms in Adult Patients. https://doi.org/10.6084/m9.figshare.28078913.v1. ^35^ This project contains the following underlying data:
1. data sheet.xlsx Data are available under the terms of the Creative Commons Attribution 4.0 International license (CC-BY 4.0). Figshare.- Correlation between Posterior Teeth Loss and Temporomandibular Joint Disorder Symptoms in Adult Patients. https://doi.org/10.6084/m9.figshare.28078913.v1. ^35^ This project contains the following underlying data:
1. symptom quastionair.pdf 2. dctmd clinical form.pdf Data are available under the terms of the Creative Commons Attribution 4.0 International license (CC-BY 4.0).

## References

[1] Okeson J : *Management of temporomandibular disorders and occlusion.* Mosby Elsevier;2013; p.129.

[2] Greene CS : The etiology of temporomandibular disorders: implications for treatment. *J. Orofac. Pain.* 2001;15(2):93–105. discussion 6-16. 11443830

[3] Arima T Svensson P Rasmussen C : The relationship between selective sleep deprivation, nocturnal jaw-muscle activity and pain in healthy men. *J. Oral Rehabil.* 2001;28(2):140–148. 10.1046/j.1365-2842.2001.00687.x 11298262

[4] Pullinger AG Seligman DA : Quantification and validation of predictive values of occlusal variables in temporomandibular disorders using a multifactorial analysis. *J. Prosthet. Dent.* 2000;83(1):66–75. 10.1016/s0022-3913(00)70090-4 10633024

[5] Carlson CR Bertrand PM Ehrlich AD : Physical self-regulation training for the management of temporomandibular disorders. *J. Orofac. Pain.* 2001;15(1):47–55. 11889647

[6] Carlsson GE Egermark I Magnusson T : Predictors of bruxism, other oral parafunctions, and tooth wear over a 20-year follow-up period. *J. Orofac. Pain.* 2003;17(1):50–57. 12756931

[7] Celic R Jerolimov V Knezovic Zlataric D : Relationship of slightly limited mandibular movements to temporomandibular disorders. *Braz. Dent. J.* 2004;15(2):151–154. 10.1590/s0103-64402004000200012 15776199

[8] Al-Jabrah OA Al-Shumailan YR : Prevalence of temporomandibular disorder signs in patients with complete versus partial dentures. *Clin. Oral Investig.* 2006;10(3):167–173. 10.1007/s00784-006-0046-3 16636843

[9] Baba K Haketa T Sasaki Y : Association between masseter muscle activity levels recorded during sleep and signs and symptoms of temporomandibular disorders in healthy young adults. *J. Orofac. Pain.* 2005;19(3):226–231. 16106716

[10] Stegenga B : Osteoarthritis of the temporomandibular joint organ and its relationship to disc displacement. *J. Orofac. Pain.* 2001;15(3):193–205. 11575190

[11] Cosme DC Baldisserotto SM Canabarro Sde A : Bruxism and voluntary maximal bite force in young dentate adults. *Int. J. Prosthodont.* 2005;18(4):328–332. 16052788

[12] Wang MQ Xue F He JJ : Missing posterior teeth and risk of temporomandibular disorders. *J. Dent. Res.* 2009;88(10):942–945. 10.1177/0022034509344387 19783804

[13] Sousa ST de Mello VV de Magalhães BG : The role of occlusal factors on the occurrence of temporomandibular disorders. *Cranio.* 2015;33(3):211–216. 10.1179/2151090314y.0000000015 25027731

[14] Javed MU Asim MA Afreen Z : Association of tooth loss with temporomandibular disorders. *Khyber Med. Univ. J.* 2020;12(1):29–33.

[15] Seligman DA Pullinger AG : Analysis of occlusal variables, dental attrition, and age for distinguishing healthy controls from female patients with intracapsular temporomandibular disorders. *J. Prosthet. Dent.* 2000;83(1):76–82. 10.1016/s0022-3913(00)70091-6 10633025

[16] Wolfart S Heydecke G Luthardt RG : Effects of prosthetic treatment for shortened dental arches on oral health-related quality of life, self-reports of pain and jaw disability: results from the pilot-phase of a randomized multicentre trial. *J. Oral Rehabil.* 2005;32(11):815–822. 10.1111/j.1365-2842.2005.01522.x 16202045

[17] Witter DJ Kreulen CM Mulder J : Signs and symptoms related to temporomandibular disorders--Follow-up of subjects with shortened and complete dental arches. *J. Dent.* 2007;35(6):521–527. 10.1016/j.jdent.2007.02.003 17400355

[18] Kopp S : Clinical findings in temporomandibular joint osteoarthrosis. *Scand. J. Dent. Res.* 1977;85(6):434–443. 10.1111/j.1600-0722.1977.tb00577.x 271341

[19] Seligman DA Pullinger AG : The role of intercuspal occlusal relationships in temporomandibular disorders: a review. *J. Craniomandib. Disord.* 1991;5(2):96–106. 1812142

[20] Sheridan SG Mittler DM Van Gerven DP : Biomechanical association of dental and temporomandibular pathology in a medieval Nubian population. *Am. J. Phys. Anthropol.* 1991;85(2):201–205. 10.1002/ajpa.1330850208 1882982

[21] Winstanley RB : A retrospective analysis of the treatment of occlusal disharmony by selective grinding. *J. Oral Rehabil.* 1986;13(2):169–181. 10.1111/j.1365-2842.1986.tb00649.x 3457134

[22] Pullinger AG Seligman DA Gornbein JA : A multiple logistic regression analysis of the risk and relative odds of temporomandibular disorders as a function of common occlusal features. *J. Dent. Res.* 1993;72(6):968–979. 10.1177/00220345930720061301 8496480

[23] Luder HU : Factors affecting degeneration in human temporomandibular joints as assessed histologically. *Eur. J. Oral Sci.* 2002;110(2):106–113. 10.1034/j.1600-0722.2002.11212.x 12013552

[24] Tallents RH Macher DJ Kyrkanides S : Prevalence of missing posterior teeth and intraarticular temporomandibular disorders. *J. Prosthet. Dent.* 2002;87(1):45–50. 10.1067/mpr.2002.121487 11807483

[25] Asendorf A Möllenkamp J Schierz O : Interexaminer reliability of the German version of the DC/TMD. *J. Oral Rehabil.* 2021 Jan;48(1):28–34. 10.1111/joor.13054 32648606

[26] Schiffman E Ohrbach R Truelove E : Diagnostic criteria for temporomandibular disorders (DC/TMD) for clinical and research applications: recommendations of the International RDC/TMD Consortium Network and Orofacial Pain Special Interest Group. *J. Oral Facial Pain Headache.* 2014;28(1):6. 10.11607/jop.1151 24482784 PMC4478082

[27] Aggarwal VR McBeth J Zakrzewska JM : The epidemiology of chronic syndromes that are frequently unexplained: do they have common associated factors? *Int. J. Epidemiol.* 2006;35(2):468–76. Epub 20051122. 10.1093/ije/dyi265 16303810

[28] Derwich M Mitus-Kenig M Pawlowska E : Temporomandibular Joints' Morphology and Osteoarthritic Changes in Cone-Beam Computed Tomography Images in Patients with and without Reciprocal Clicking-A Case Control Study. *Int. J. Environ. Res. Public Health.* 2020;17(10). Epub 20200514. 10.3390/ijerph17103428 32423066 PMC7277452

[29] Schokker RP Hansson TL Ansink BJ : Craniomandibular disorders in patients with different types of headache. *J. Craniomandib. Disord.* 1990;4(1):47–51. 2098387

[30] Calixtre LB Oliveira AB Sena Rosa LR de : Effectiveness of mobilisation of the upper cervical region and craniocervical flexor training on orofacial pain, mandibular function and headache in women with TMD. A randomised, controlled trial. *J. Oral Rehabil.* 2019;46(2):109–119. 10.1111/joor.12733 30307636

[31] Kitsoulis P Marini A Iliou K : Signs and symptoms of temporomandibular joint disorders related to the degree of mouth opening and hearing loss. *BMC Ear Nose Throat Disord.* 2011;11:5. Epub 20110525. 10.1186/1472-6815-11-5 21612586 PMC3117795

[32] Sawair FA Hassoneh YM Al-Zawawi BM : Maximum mouth opening. Associated factors and dental significance. *Saudi Med. J.* 2010;31(4):369–373. 20383412

[33] Mundt T Mack F Schwahn C : Gender differences in associations between occlusal support and signs of temporomandibular disorders: results of the population-based Study of Health in Pomerania (SHIP). *Int. J. Prosthodont.* 2005;18(3):232–239. 15945311

[34] Shaw RM Molyneux GS : The effects of mandibular hypofunction on the development of the mandibular disc in the rabbit. *Arch. Oral Biol.* 1994;39(9):747–752. 10.1016/0003-9969(94)90003-5 7802610

[35] Hussein Ayaz B : Correlation between Posterior Teeth Loss and Temporomandibular Joint Disorder Symptoms in Adult Patients.Dataset. *figshare.* 2024. 10.6084/m9.figshare.28078913.v2 PMC12120414 40443911

