# Supplementary material for: Correlation between posterior teeth loss and temporomandibular joint disorder symptoms in adult patients
Source: F1000Res. 2025 Apr 11;14:428. [Version 1] doi: 10.12688/f1000research.161006.1 (PMC12120414; doi:10.12688/f1000research.161006.1)
Supplement: Peer review history

# Reviewer report: Reviewer response for version 1

**DOI:** 10.5256/f1000research.176980.r378143

**Published:** 2025-05-28

**Authors:** Nancy Ajwa

**Affiliations:** 1 College of Dentistry, Riyadh Elem University, Riyadh, Saudi Arabia

**Title and Abstract**

1. **Title Suggestion**: Consider slightly refining the title to specify the study design (e.g., *"A cross-sectional study on the correlation between posterior teeth loss and temporomandibular joint disorder symptoms in adult patients"*), which can enhance clarity and attract the appropriate readership.
2. **Abstract Clarity**:
  - Add brief details on the inclusion/exclusion criteria in the "Methods" section.
  - Include a numeric summary of key findings (e.g., prevalence, odds ratios) for better impact.

**Introduction**

1. **Literature Context**:
  - Strengthen the rationale by briefly elaborating on **conflicting evidence** in the literature and how this study fills a gap.
  - Include more recent references from the last 5 years to show up-to-date knowledge.
2. **Objectives**:
  - Clearly define a **primary hypothesis or objective** at the end of the introduction.

**Methods**

1. **Sampling Technique**:
  - Mention how the **convenience sampling** may influence generalizability and briefly justify its use.
2. **DC/TMD Protocol**:
  - Provide a **reference or footnote** for the Arabic translation validation of the DC/TMD Axis I questionnaire, if available.
3. **Calibration**:
  - Include the **intra-examiner reliability score** (e.g., kappa value) if calculated, to support reproducibility.
4. **Ethics**:
  - The IRB and consent details are clear — good inclusion.

**Results**

1. **Tables**:
  - Some table captions (e.g., Tables 1–6) could include a brief description of what comparison is being made for easier interpretation.
2. **Presentation**:

- Consider **graphical representations** (e.g., bar charts or box plots) for key variables like "maximum mouth opening" and "number of missing teeth" to enhance visual impact.

1. **Statistical Detail**:

- Report **confidence intervals** for all significant findings in-text, not just in regression tables.

**Discussion**

1. **Interpretation**:

- Consider discussing possible **confounders or effect modifiers** (e.g., bruxism, prosthesis use) that may have influenced results but were not analyzed.

1. **Clinical Relevance**:

- Include a sentence or two on **how these findings could impact clinical practice**, particularly prosthodontic or orofacial pain management.

1. **Limitations**:

- Expand briefly on **selection bias** due to convenience sampling and possible **self-report bias** in questionnaire responses.

**Conclusion**

1. **Conciseness**:

- Slightly rephrase the conclusion to be more **actionable**—e.g., “Clinicians should consider posterior tooth loss as a potential contributing factor to TMD symptoms.”

**Other Minor Suggestions**

1. **Language & Grammar**:

- Perform a **language check or proofreading** pass, especially for punctuation, article usage, and syntax to improve readability.

1. **Reference Formatting**:

- Ensure all references conform to the journal’s citation style (e.g., spacing, punctuation).

**Final Comment to Authors**

 The manuscript presents important findings on the relationship between posterior teeth loss and TMD symptoms, which has clinical value. With minor revisions as noted above to improve clarity, detail, and presentation,

Is the work clearly and accurately presented and does it cite the current literature?

Yes

If applicable, is the statistical analysis and its interpretation appropriate?

Yes

Are all the source data underlying the results available to ensure full reproducibility?

Yes

Is the study design appropriate and is the work technically sound?

Yes

Are the conclusions drawn adequately supported by the results?

Yes

Are sufficient details of methods and analysis provided to allow replication by others?

Yes

Reviewer Expertise:

Public Health

I confirm that I have read this submission and believe that I have an appropriate level of expertise to confirm that it is of an acceptable scientific standard.

# Reviewer report: Reviewer response for version 1

**DOI:** 10.5256/f1000research.176980.r378136

**Published:** 2025-05-20

**Authors:** Eman AlJoghaiman

**Affiliations:** 1 Imam Abdulrahman Bin Faisal University, Dammam, Saudi Arabia

The manuscript presents a valuable investigation into the correlation between posterior tooth loss and TMJ disorder symptoms. While the study is well-structured and addresses an important clinical question, the following constructive comments are offered to enhance clarity, interpretation, and rigor:

1. **Study Design and Limitations:**
  - The cross-sectional design limits causal inference. Please explicitly acknowledge this in the discussion and clarify that findings represent associations, not causation.
  - The convenience sampling may affect generalizability and statistical power. Consider discussing these as limitations and recommending larger, randomized studies for validation.
2. **Diagnostic Methodology:**
  - Clarify whether imaging (e.g., MRI) was used to confirm disc displacement diagnoses. Reliance on clinical examination alone may introduce diagnostic bias; this should be noted as a limitation.
  - Address the absence of psychosocial factors (DC/TMD Axis II) in the results. If these data were collected, include a brief summary; if not, justify their exclusion.
3. **Questionnaire Validation:**
  - Confirm the validity and reliability of the Arabic-translated DC/TMD questionnaire. If validation studies exist, cite them; otherwise, acknowledge this as a potential limitation.
4. **Mechanistic Discussion:**
  - Elaborate on potential biomechanical mechanisms linking posterior tooth loss to TMJ disorders (e.g., altered condylar position, overloading). This would strengthen the discussion by contextualizing findings within existing pathophysiological frameworks.
5. **Statistical Considerations:**
  - The wide confidence intervals in logistic regression models (e.g., OR=6.089, CI: 0.071–525.156) suggest imprecision due to sample size. Discuss this as a limitation.
6. **Clinical and Demographic Factors:**
  - Discuss whether confounding variables (e.g., parafunctional habits, occlusal contacts post-tooth loss) were assessed. If not, propose their inclusion in future studies.
7. **Language and Conclusions:**
  - Avoid implying causation (e.g., "posterior tooth loss *leads to* TMJ changes"). Use correlational language consistently.
  - The abstract’s conclusion could be tempered to align with the study’s observational design.
8. **Ethical and Methodological Rigor:**
  - Reaffirm that informed consent procedures adhered to ethical standards, though this is likely implicit.

**Summary:**

 The manuscript contributes meaningful insights into TMD and posterior tooth loss. Addressing the above points, particularly regarding limitations, diagnostic methods, and mechanistic context, will strengthen its impact and clarity.

Is the work clearly and accurately presented and does it cite the current literature?

Yes

If applicable, is the statistical analysis and its interpretation appropriate?

Yes

Are all the source data underlying the results available to ensure full reproducibility?

Yes

Is the study design appropriate and is the work technically sound?

Yes

Are the conclusions drawn adequately supported by the results?

Yes

Are sufficient details of methods and analysis provided to allow replication by others?

Yes

Reviewer Expertise:

Dentistry

I confirm that I have read this submission and believe that I have an appropriate level of expertise to confirm that it is of an acceptable scientific standard.
